# NRF2 immunobiology in cancer: implications for immunotherapy and therapeutic targeting

**DOI:** 10.1038/s41388-025-03560-4

**Published:** 2025-09-13

**Authors:** Harit Panda, Natalie G. Rowland, Caroline M. Krall, Brittany M. Bowman, Michael B. Major, Paul Zolkind

**Affiliations:** 1 Department of Otolaryngology, Washington University School of Medicine, St. Louis, MO USA; 2 Department of Biological Chemistry, Center for RNA Biomedicine, Rogel Cancer Center, University of Michigan Medical School, Ann Arbor, MI USA; 3 Department of Cell Biology and Physiology, Washington University School of Medicine, St. Louis, MO USA

**Keywords:** Cancer microenvironment, Cancer models

## Abstract

Nuclear factor erythroid 2-related factor 2 (NRF2) is a transcription factor that acts as a key regulator in cellular defense mechanisms against oxidative stress and xenobiotics. NRF2 modulates the expression of over 200 genes involved in antioxidant response, drug metabolism, and cellular resilience. Constitutive activation of NRF2 is a common event in cancer and recent advances provide remarkable insights into the role of NRF2 in oncogenesis, immune evasion, and treatment resistance. This review aims to provide a comprehensive overview of the role of NRF2 in shaping the tumor immune microenvironment and the impact this has on clinical outcomes and treatment opportunities. Across multiple tumor subtypes, the activation of NRF2 is associated with impaired responses to anti-PD1 immunotherapy. Mechanistic insights from genetically engineered mouse models, in vitro studies, and clinical trial samples demonstrate how NRF2 activity supports cell resiliency, diminishes cytotoxic immune responses, and promotes metabolic reprogramming. This also provides a vulnerability which can be targeted through novel drug therapy and future directions will include development of optimal combination strategies to target tumor dependencies while minimizing toxicity and systemic off-target immune related effects.

## Introduction

The toxic accumulation of reactive oxygen species (ROS) is damaging to cells and contributes to a range of human diseases. To neutralize ROS and other electrophilic stressors, various cytoprotection mechanisms have evolved. Key among these is the acute transcriptional activation of genes harboring CNC-sMaf binding elements (CsMBE), many of which encode cytoprotective proteins that neutralize free radicals and limit cellular damage. In 1994, Yuet Kan’s lab discovered a powerful transcriptional activator of the CsMBE and the cellular REDOX response—a cap’n’collar (CNC) protein they named NRF2 (also known as NFE2L2) [1]. Over the past three decades, our understanding of NRF2 regulation and its importance in health and disease has increased dramatically. In addition to mitigating oxidative stress, a growing body of literature establishes key roles for NRF2 in metabolic reprogramming and the immune microenvironment. Across many cancer types, aberrant, constitutively active NRF2-dependent transcription promotes tumor initiation, progression, and therapeutic resistance, and ultimately portends poor clinical outcomes. Although highly sought and with numerous candidates under development, NRF2 inhibitors are not yet available in the clinic. In this review, we present a synthesis of the NRF2 literature with an emphasis on the role of NRF2 in the tumor immune microenvironment and in controlling the response to immunotherapy.

## NRF2 activation mediates the cellular response to stress

**Fig. 1:**
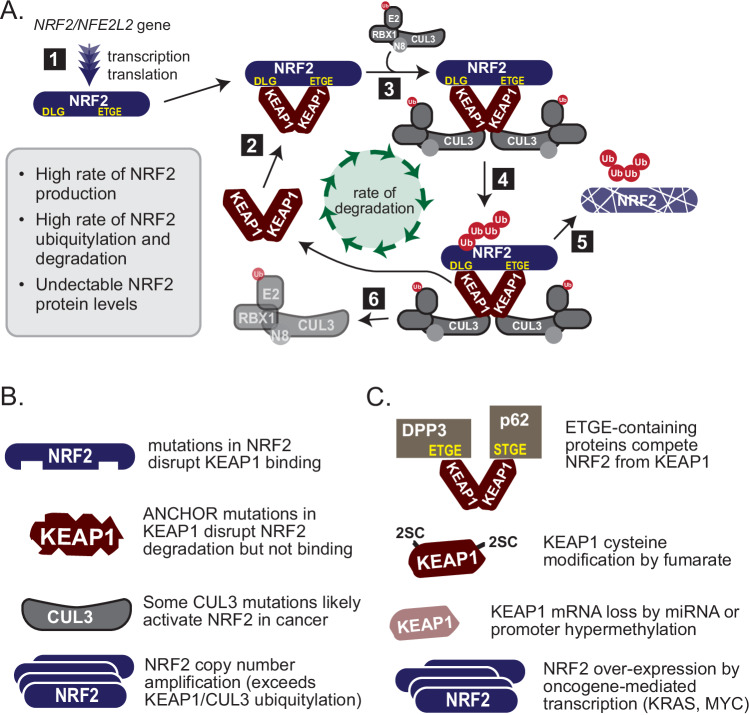
Mechanistic overview of NRF2 regulation. **A** Post-translational control dominates NRF2 regulation across tissue types [1]. The NFE2L2/NRF2 gene is constitutively transcribed and translated [2]. Subsequent to its translation, homodimeric KEAP1 binds the NRF2 protein with high affinity [3, 4]. Given the low binding affinity between KEAP1 and CUL3, it likely that two CUL3 proteins bind KEAP1 after KEAP1:NRF2 co-complex, resulting in NRF2 ubiquitylation [5, 6]. Ubiquitylated NRF2 is then released to the proteasome for degradation simultaneous with release of CUL3 from KEAP1 via the CAND1/2-neddylation cycle. Ultimately, in normal cells not experiencing stress, the rate of this ubiquitylation cycle is envisioned to match the rate of NRF2 production resulting in undetectable levels of NRF2 protein. It is clear that some electrophiles slow the NRF2 degradation cycle by decreasing the KEAP1:CUL3 interaction. **B** Genomic mechanisms of NRF2 activation in cancer include NRF2 hotspot mutations, KEAP1 inactivating mutations, CUL3 inactivating mutations, and NRF2 copy number amplifications. **C** Non-genomic mechanisms also activate NRF2 in cancer, including KEAP1 cysteine modifications by fumarate and competitive displacement of NRF2 from KEAP1 through the binding of proteins with an ETGE or ETGE-like motif.

The NRF2 transcription factor is a critical mediator of cellular redox homeostasis. In the absence of stress or pathway alteration, NRF2 protein levels are kept low by the Kelch-like-ECH-associated-protein 1 (KEAP1) and cullin3 (CUL-3) protein complex (Fig. 1A) [2]. KEAP1 functions as a substrate recognition adaptor for a CUL3 scaffolded E3 ubiquitin ligase complex. KEAP1 is an obligate homodimer that binds one molecule of NRF2 through the NRF2 ^79^ETGE and ^29^DLGex motifs. A bound KEAP1:NRF2 complex is inactive until it engages two molecules of CUL3 to create a CUL3:KEAP1:NRF2 complex at a 2:2:1 stoichiometry. Lysine residues positioned between the DLG and ETGE motifs in NRF2 become ubiquitylated, resulting in NRF2 proteasomal degradation and a ~ 15 min half-life [2].

KEAP1 contains 27 cysteine residues that have been stratified based upon their reactivity to various electrophiles [3]. Oxidative and electrophilic stress modifies these cysteines in KEAP1, ultimately suppressing KEAP1:CUL3-dependent degradation of NRF2. The mechanism(s) by which specific electrophiles and their cognate cysteines impedes NRF2 degradation is actively being studied; some electrophiles decrease the KEAP1:CUL3 association. In the absence of a fully catalytic KEAP1:CUL3 complex, newly transcribed and translated NRF2 is free to translocate into the nucleus where it heterodimerizes with small Maf proteins (sMAF) [4]. sMaf proteins function as obligatory partners for CNC bZip proteins, including NRF2 [5]. The NRF2-sMAF complex binds to the antioxidant response element (ARE, also referred to as CsMBE) within the promoters of its target genes. Once bound, the NRF2-sMAF complex promotes the expression of genes that govern four distinct but interconnected processes within the cell: (1) antioxidant response, (2) drug detoxification, (3) cellular metabolism, and (4) inflammation [2]. Activation of the antioxidant response is considered a primary and evolutionarily conserved function for NRF2. For example, NRF2 transcriptional activation of glutamate-cysteine ligase catalytic subunit (GCLC) and glutamate-cysteine ligase modifier subunit (GCLM) increases the production of the ROS scavenger γ-glutamyl-cysteinyl glycine, more commonly referred to as glutathione (GSH) [6]. Other NRF2-transcriptionally regulated genes with important antioxidant function include glutathione peroxidase 2 (GPX2) and thioredoxin reductase 1 (TXNRD1) [2].

## Hallmarks of NRF2 activation in cancer

There are several mechanisms by which NRF2 becomes constitutively activated in cancer cells (Fig. 1B, C) [7]. Gain of function mutations in NRF2 and loss of function KEAP1 and CUL3 mutations have been identified in multiple solid tumor types [8]. Mutations in the DLGex and ETGE motif of NRF2 impair NRF2 binding to the KEAP1 homodimer [9]. ANCHOR mutations in KEAP1 are hypomorphic for NRF2 degradation but retain NRF2 binding. Several miRNA including miR-28 and miR-144 are integral to the activity and regulation of NRF2. miRNA which target both NRF2 and KEAP1, referred to as redox miRs, have demonstrated involvement in promoting carcinogenesis, radiation and cisplatin resistance [8]. Additionally, several commonly dysregulated cancer signaling pathways have been associated with NRF2 activation including *BRAF, RAS-RAF-MAPK, Myc, and p53* [10–12].

High NRF2 activity is found predominantly in cancers associated with carcinogen exposure including non-small cell lung cancer (NSCLC), esophageal cancer, endometrial carcinoma, bladder cancer, and head and neck cancers [13]. NRF2 pathway activation provides a survival advantage to cancer cells through supporting several important cancer programs. For example, NRF2-active tumors have an enhanced ability to withstand stressors that would typically induce cellular apoptosis. NRF2 binds the ARE on the gene promoter region of the anti-apoptotic proteins, Bcl-2 and Bcl-xL, induces their expression, directly enhancing cell survival during cytotoxic stress [12, 14]. Other mechanisms to avoid apoptosis include via the homeodomain-interacting protein kinase 2 (HIPK2) feedback loop which supports anti-apoptotic/pro-survival function during hypoxic stress and DNA damage [15]. Relatedly, NRF2 targets, SCL7A11, HMOX1 and GPX4, have been implicated in inhibiting ferroptosis, a unique mechanism of cell death associated with an increase in free iron and the accumulation of lipid peroxides [16].

NRF2 may also be associated with enhanced invasion and migration potential including the promotion of epithelial-to-mesenchymal transition, through loss of E-cadherin and enhanced Notch signaling [17]. Across multiple cancer types, there is conflicting evidence with some studies reporting that NRF2 signaling promotes EMT targets including Snai1, N-Cadherin, ZEB-1 [17, 18]. Using spatial transcriptomics and single cell sequencing of MC38 tumor cells in mice treated with agonist anti-CD40 antibody therapy, it was demonstrated that heme-driven NRF2 activation in macrophages drives metabolic reprogramming towards immunosuppressive tumor associated macrophages (TAMs) [19]. These NRF2-high TAMs stabilized an EMT phenotype in malignant cells and promoted treatment resistance [19].

The development of KEAP1 and NRF2 knockout and conditional knockout GEMMs has significantly enhanced our understanding of KEAP1 and NRF2 function in both tumorigenesis and immunosurveillance (See Table 1 for references). Thus far, there is no evidence to suggest that hyperactivation of NRF2 alone results in the spontaneous development of tumors. An abundance of literature, however, supports that NRF2 activation promotes increased tumor initiation, progression and metastases. A summary of phenotypes for KEAP1 and NRF2 mutant mice is provided in Table 1. For example, Kras^G12D/+^;Keap1^fl/fl^ lung adenocarcinoma GEMM demonstrated twice the number of lung tumors relative to Kras^G12D/+^ mice, and were also associated with a significantly restricted expansion of infiltrating macrophages [20]. Similarly, Keap1^fl/fl^;Stk11^fl/fl^;Kras^LSL-G12D^ mice developed earlier onset and greater volume of tumor burden than in non-Keap1^fl/fl^ mice [21]. Interestingly, a study by DeBlasi et al. using the Kras^G12D/+^; p53^fl/fl^ (KP) lung adenocarcinoma model found that the addition of NRF2 activation via Keap1^R554Q/R554Q^ or Nrf2^D29H/+^ supported tumor initiation and progression of lesions into early stage (I and II) cancers but also identified a notable decrease in the burden of grade 3 and 4 tumors [22].

**Table 1:** NRF2-active genetically engineered mouse models.

Genotype	*Keap1*	*Nfe2l2*	Phenotype	Reference
*Keap1*^*flox/flox*^*::* *Trp53*^*flox/flox*^*::* *R26*^*tdTomato*^	CKO	WT	- Keap1/p53 deletion in airway basal stem cells produce lung SCC. - KEAP1 deletion increased tumor aggressiveness, metastasis, and resistance to radiotherapy	Jeong *Cancer Discovery 2017*
*Kras*^*LSL-G12D/+*^*::* *Tp53*^*flox/flox*^ with *sgKeap1*	KO	WT	- Mouse model of LUAD - Increased tumor burden and aggressiveness	Romero *Nature medicine 2017*
*Keap1*^*fl/fl*^ *Pten*^*fl/fl*^	CKO	WT	- No phenotype with Keap1 loss alone - Combined loss of *Keap1* and *Pten* produces lung adenocarcinoma	Best Cell Metabolism 2018
*Kras*^*LSL-G12D/+*^ *p53*^*fl/fl*^ *Keap1-Intratracheal Crispr KO*	CKO	WT	- Intratracheally lentiviral delivery produced LUAD - Increased metastatic phenotype in KEAP1ko tumors - NRF2 activation promoted enhanced expression of Bach1	Lignitto Cell 2019
*KrasG12D* *Keap1*^*fl/fl*^	CKO	WT	- KEAP1 deletion increased number of LUAD tumors of bronchiolar cell-of-origin - Reduced inflammatory response in KEAP1-KO tumors	Best Nature Communications 2019
*Kras*^*LSL-G12D/+*^ *p53*^*fl/fl*^ *Keap1*^*fl/fl*^ *Slc33a1-KO*	CKO	WT	- KEAP1-KO increased high-grade adenocarcinomas, tumor burden, tumor frequency - SLC33a1-KO decreased tumor burden and reduced number of high grade LUADs	Romero Nature Cancer 2020
*LoxP-Stop-loxP Kras*^*G12D*^ *Keap1*^*FB/FB*^ *Keap1*^*FA/FA*^	CKO	WT	- NRF2 activation in Kras-driven tumor cells promoted tumor growth and reduced survival - Activated host NRF2 in microenvironment suppressed tumor burden and prolonged survival	Hayashi Cancer Research 2020
*Kras*^*LSL-G12D*^ *Stk11*^*fl/fl*^ *Keap1*^*fl/fl*^	CKO	WT	*- Keap1*^*fl/fl*^*Stk11*^*fl/fl*^*Kras*^*LSL-G12D*^ mice demonstrate early, multifocal lung tumor development, mice develop progressive lung failure	Singh Clinical Cancer Research 2021
*Trp53*^*fl/fl*^ *p16*^*fl/fl*^ *LSL-Nrf2*^*E79Q/+*^	WT	Transgene	- Mouse model of C-SCLC and P-SCLC. NRF2 silenced in C-SCLCs. Higher incidence of P-SCLCs in Nrf2E79Q/+ mice	Hamad Oncogene 2022
*Kras*^*G12D/+*^ *Keap1*^*R554Q*^ *Nrf2*^*D29H*^	Conditional transgene	Conditional transgene	- Keap1^R554Q/R554Q^ and Nrf2^D29H/+^ increased NSCLC tumor number in Kras^G12D/+^ model but disproportionately increased Grade 1 tumors and reduced Grade 3 tumors	DeBlasi Cancer Research 2023
*Kras*^*G12D/+*^ *p53*^*fl/fl*^ *KEAP1*^*fl/fl*^	Conditional transgene	WT	- KEAP1^+/fl^ tumors grew faster - Keap-KO associated with diminished immune responses	Zavitsanou Cell Reports 2023
*NRF2*^*L30F*^ *TRP53*^*R172H*^ *Keap1*^*-/-*^	KO	Transgene	- NRF2^L30F^TRP53^R172H^ but not TRP53^R172H^Keap1^-/-^ mice develop esophageal squamous cell carcinoma	Takahashi Cell Reports 2024
p16^INK4A^ p53^-/-^ NRF2^E79Q^	WT	Conditional Transgene	- p16^INK4A^p53^–/–^NRF2^E79Q^ but not p16^INK4A^p53^-/-^NRF2^+/+^ mice develop oral squamous cell carcinoma	Hamad Cancer Research Communications 2024

NRF2-active genetically engineered mouse oncology models. Conditional knockout (CKO), knockout (KO), Wild-type (WT), squamous cell carcinoma (SCC), lung adenocarcinoma (LUAD), combined small cell lung cancer (C-SCLC), pure small cell lung cancer (P-SCLC)

## NRF2 prevents cytotoxicity from immune system

The human immune system operates via two main strategies: an innate immune response that recognizes general hallmarks of invading substances and an adaptive immune response that targets specific antigens. In general, the innate response serves as the front line of defense for the body while the adaptive response is much more specialized. Despite its lack of specificity, the innate immune system comprises a multitude of differentiated cell types that have evolved to protect the body from foreign invaders and have developed complex responses to pathogens, toxins, and allergens [23]. These effector cells, such as phagocytes, NK cells, and innate lymphoid cells, trigger an acute phase response characterized by nonspecific host defenses to promote inflammatory signaling, vascular permeability, complement factors, and cytokine production including IL-1, IL-6, TNF-α [24]. The effector phagocytes of the innate immune response are capable of combatting microorganisms through an array of microbicidal enzymes as well as the production of ROS and ROI [24]. The production of ROS and ROI by neutrophils is predominantly through the NADPH oxidase, NOX2, which is activated by inflammatory proteins including TNF, GM-CSF, insulin, platelet derived growth factor, among others [25]. This is a vital function of the innate immune system, and individuals with congenital NOX2 deficiency suffer from chronic granulomatous disease, characterized by recurrent and severe infections [26]. One critical function of the immune system, however, is to balance the need for rapid and effective pathogen elimination with the requisite to minimize collateral damage to healthy tissue.

The NRF2 pathway is activated in the setting of acute inflammation and functions to reduce excessive tissue damage by tampering the acute cytotoxic effects of the innate immune response. The most direct evidence of the role of NRF2 in regulating immune responses is from studies of NRF2-deficient (NRF2 KO) mice which exhibit profound and destructive injury in the setting of immune responses. NRF2 KO mice have been shown across numerous studies to have increased susceptibility to the detrimental effects of unmitigated cytotoxic inflammation. In a model of LPS-induced sepsis, NRF2 KO mice demonstrated significant increases in neutrophil ROS generation, pro-inflammatory cytokines and chemokines, and decreased mortality [27]. Similar studies have demonstrated NRF2 KO mice have increased susceptibility to autoimmune conditions such as lupus nephritis [28] as well as pulmonary [29] and cardiovascular disease [30]. Mice deficient in the NRF2 target gene NQO1 also exhibit decreased apoptosis in thymocytes and a predisposition to autoimmune arthritis [31]. Therefore, NRF2 serves as a critical host defense to balance the body’s need for innate immune responses against toxins and invasive pathogens, with the individual cell’s interest in self-preservation. As a result, NRF2 activators have proven to be an area of active clinical interest in the context of autoimmunity with the goal of enhancing cellular defenses against aberrant immune-related destruction.

### Mechanisms of NRF2-mediated immunosuppression

**Fig. 2:**
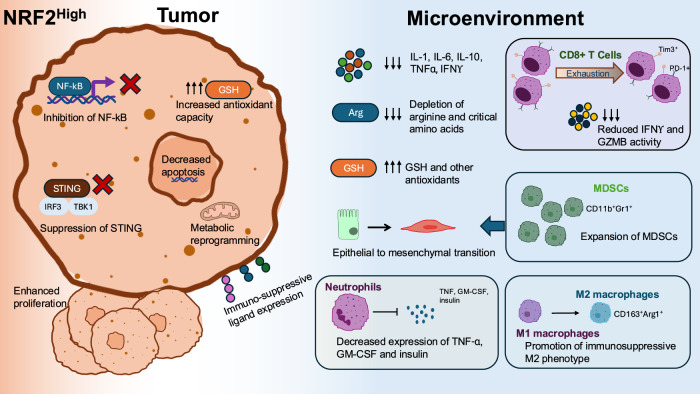
Implications of high NRF2 signaling in tumor and immune cells within the tumor microenvironment. Tumor-intrinsic activation of NRF2 (left) leads to cellular reprogramming to promote immunoevasion and tumor resilience, including suppression of NF-κB signaling, downregulation of the STING pathway, expression of immunosuppressive ligands, and enhanced metabolic reprogramming. In the immune microenvironment (right), NRF2 signaling alters both myeloid and lymphoid compartments. Myeloid populations show expansion of CD11b⁺Gr1⁺ myeloid-derived suppressor cells (MDSCs), increased polarization of macrophages toward an M2-like phenotype, and reduced secretion of TNF-α, GM-CSF, and insulin by neutrophils. Lymphoid cells, particularly CD8 T cells, exhibit functional exhaustion with decreased IFN-γ and granzyme B expression. These microenvironmental changes collectively suppress pro-inflammatory cytokine production (IL-1, IL-6, IL-10, TNF-α, IFN-γ), and deplete essential nutrients such as amino acids (center).

Despite robust literature from NRF2 KO mice supporting the role of NRF2 on the immune system, the mechanisms underlying this phenotype are numerous and remain an active area of research (Fig. 2). The most well-established mechanisms include through (1) NF-κB inhibition, (2) suppression of STING signaling, (3) interference of pro-inflammatory cytokine transcription, and (4) through NRF2 target gene signaling pathways. Evidence supports that NRF2 mediated immune suppression developed in part to reduce tissue injury from NF-κB signaling. NF-κB is a transcription factor family activated by pro-inflammatory cytokines including TNFα and IL-1*B* with a critical role in regulating both innate and adaptive immune responses, lymphocyte differentiation and proliferation, and cellular stress responses [32]. NRF2 and NF-κB are similarly regulated by oxidative and electrophilic stress and, therefore, this balance is critical for maintaining appropriate inflammatory responses. NF-κB signaling is significantly heightened in NRF2 KO mice models [27] and this is associated with increased inflammatory cytokine production [33]. NRF2 activation with sulforaphane is associated with inhibition of NF-kB binding and transcriptional activity, supporting the existing a direct cross-talk mechanism between the two pathways [34]. Likewise, the p65 subunit of NF-κB has been demonstrated to directly reduce NRF2 transcriptional activity through its interaction with KEAP1, activating NRF2 transcriptional repressors and sequestration of coactivators such as CREB binding protein [35].

NRF2 activity also has a suppressive effect on the Stimulator of Interferon Genes (STING) pathway. STING, a critical cellular defense in promoting immune resistance to microbial pathogens and cancers, is activated by the presence of cytosolic DNA or cyclic dinucleotides (CDNs) within the cell cytoplasm [36]. Activation of STING induces a downstream pathway consisting of TBK1 and IRF3 which culminates in the activation of NF-κB and induction of type I IFN responses [37]. Studies in human epithelial cell lines, fibroblasts, and primary human monocyte-derived macrophages identified that NRF2 activation was directly inhibitory of STING and its downstream pathway, a finding that was reversed with NRF2 inhibition [38]. Mechanistically, NRF2 regulation of STING is controlled post-transcriptionally by impairing STING mRNA stability [38]. Similarly, loss of KEAP1 promotes stabilization and accumulation of EMSY, a proto-oncogene typically targeted by KEAP1 for degradation [39]. Accumulation of EMSY in KEAP1-mutant tumors suppresses type I interferon responses and promotes immune evasion, a phenotype which can be reversed with the STING agonist, DMXAA [39]. There are important clinical implications of this inhibitory relationship as STING agonists have been widely explored for use as immunotherapy sensitizing agents but have not reached clinical approval. In a novel therapeutic approach utilizing NRF2-specific small interfering RNA (siRNA) delivered in combination with laser irradiation, there is pronounced induction of phosphorylated STING signaling [40]. This combination approach enhanced cytotoxic immune responses characterized by increased expression of IFNγ, TNFα, and IL-10 [40].

Post-transcriptional regulation of STING by NRF2 stands in contrast to other immunosuppressive functions of NRF2 which are controlled at the transcriptional level. For example, a recent chromatin immunoprecipitation (ChIP)-seq and ChIP-qPCR analysis demonstrated NRF2 binds to the upstream regions of pro-inflammatory cytokine genes, IL-6 and IL-1b and has a suppressive effect on pro-inflammatory (M1) activated macrophages [41]. In a related study, macrophages co-cultured with NRF2 active cancers were driven to an immunosuppressive M2 phenotype characterized by increased CD163 and Arg1 as well as diminished IL-6 and IL-1b [42]. Furthermore, genetically engineered mouse model (GEMM) studies of KEAP1-KO mice with constitutively active NRF2 demonstrate marked expansion of myeloid derived suppressor cells (MDSCs) characterized by CD11b^+^Gr-1^+^, arginase expression and suppression of T cell activity [43]. Myeloid derived suppressor cells support tumor growth and T cell inhibition through multiple mechanisms including secretion of inhibitory cytokines, production of ROS which inhibit T cell activation, and depletion of arginine and critical amino acids in the tumor microenvironment [44]. Evidence from NRF2^+/+^ and NRF2^-/-^ GEMMs demonstrate that MDSCs also utilize NRF2 to withstand and further propagate an oxidative environment toxic to T cells and NK cells [45]. Interestingly, the presence of NRF2-active MDSC population, systemically, may suppress cancer metastases, a phenotype reversed in NRF2-deficient mice [46]. This supports multiple prior studies demonstrating that chemical carcinogenesis is enhanced in NRF2-absent models [47]. This is also consistent with the long understood paradox that NRF2 deficiency may increase susceptibility to carcinogenesis whereas increased NRF2 activity in an already developed tumor supports many of the hallmarks of cancer progression and survival [48].

In addition to the NRF2-specific mechanisms mentioned above, much of the NRF2-mediated immune regulation is carried out through NRF2 target genes. For example, NQO1 also inhibits cytotoxic cytokines including IL-6, IL-1B, and TNFα through TLR suppression in macrophages and microglia, a finding with clinical significance in both cancers and neurodegenerative disorders [49]. Likewise, HMOX1-1 which is predominantly regulated by NRF2 and has a critical role in heme degradation and mediating ferroptosis, also possesses binding sites for NF-κB and AP-1 within its promoter region [50]. HMOX-1 has been identified as an immunoregulator of macrophage differentiation, promoting an anti-inflammatory, IL-10 secreting M2 phenotype [50]. Data from in vitro studies and in HMOX1 − /− mice support that HMOX1 binds STAT3, inhibits T_H_17 differentiation, and promotes the differentiation of regulatory T cells (T_regs_) [50].

## NRF2 and resistance to genotoxic and targeted therapy

NRF2 activity has been associated with resistance to chemotherapy, radiation and immunotherapy [12, 48, 51–54]. The burden of NRF2-mediated resistance is significant and, with no NRF2 inhibitors commercially available, the impact on patient outcomes is substantial. The effects of NRF2 pathway activation on treatment resistance have been demonstrated across multiple tumor types in both in vitro and in vivo studies. For example, NRF2-active DU-145 prostate cancer cells and TGBC24TKB gallbladder cancer cells demonstrate chemotherapy resistance, in part due to impaired drug clearing and detoxification [55]. Relatedly, Oren et al. demonstrated that a subset of PC9 lung cancer cells can adopt an NRF2-active program in response to osimertinib, a third-generation EGFR tyrosine kinase inhibitor, characterized by enhanced glutathione metabolism, antioxidative response, and a shift to fatty acid metabolism [56]. This subset defined cycling persister cells which maintain proliferative capacbilities even in the setting of cytotoxic chemotherapy [56].

The translational relevance of these finding is demonstrated in retrospective studies examining genomic differences in tumors responsive and resistant to treatment. Across a cohort of NSCLC patients, those with KEAP1/NRF2 mutant tumors had a nearly 60% rate of locoregional failure after chemoradiation relative to 20% in the KEAP1/NRF2 wildtype cohort [51]. A related investigation in stage IV NSCLC identified profound outcome differences in patients harboring NRF2/KEAP1/CUL3 mutant tumors treated with first line chemotherapy [52]. In the NRF2/KEAP1/CUL3 mutant cohort time to failure (TTF) was 2.8mo and overall survival (OS) 11.2mo, compared to 8.3mo (TTF) and 36.8mo (OS) in the control cohort [52]. Two studies in head and neck squamous cell carcinoma (HNSCC) have demonstrated associations between NRF2 activation and clinical outcomes. Namani et al. applied a 17-gene NRF2 signature to four independent HNSCC datasets and revealed significantly worse OS in the NRF2-active cohorts compared to the control cohort [53]. A slightly refined, 10-gene KEAP1/NRF2/CUL3 signature applied to the same dataset produced a similarly striking difference in overall survival and also identified that cisplatin resistant HNSCC tumors adopt an NRF2-active program to improve suvival and proliferative capacity ^51^. Related studies have shown NRF2 active tumors portend a worse prognosis across numerous cancer subtypes including esophageal, breast, brain, gastric and colorectal cancers [57].

## Tumor-intrinsic NRF2 signaling promotes immunoevasion

The tumor immune microenvironment is a complex, heterogenous system responsive to many different factors including cytokines, chemokines, neurotrophic and morphogenic factors, and others which regulate the chemotaxis and activity of immune cells within tumors and draining lymph nodes. The immune system is integrally involved in the initiation and development of tumors, where malignant cells face constant immune pressure to evolve suppressive and evasive mechanisms to prevent immune mediated eradication [58]. A confluence of recent studies have proven that constitutive NRF2 activity in tumor cells impacts the immune microenvironment with important consequences on treatment responsiveness and therapeutic vulnerability. A pan-cancer analysis demonstrated that tumors with genetic activation of NRF2 exhibit qualities of enhanced immunoevasion including enrichment of MHC-I mutations, reduced immune infiltration, and suppression of activating ligands for NK cells [59]. Likewise, an elegant study utilizing a Kras^G12D/+^; p53^−/−^;KEAP1^R470C^ with a Y chromosome-driven model antigen introduced into female immunocompetent hosts revealed enhanced tumor growth relative to KEAP1-wildtype tumors [60]. This immunoevasive phenotype is mediated in part through diminished dendritic cell and T cell responses, a finding that was also recognized in human lung adenocarcinoma patient samples [60]. A similar finding was noted in a single cell RNA-seq analysis of lung adenocarcinomas which demonstrated KEAP1 mutation was associated with increased TAMs and diminished cytotoxic T cells [61].

The finding of an NRF2-associated “immune cold” phenotype extends beyond non-small cell lung cancers and has significant clinical implications. An in vivo mouse experiment using the syngeneic mouse oral cancer line (MOC1) with and without activating NRF2^E79Q^ mutations demonstrated that NRF2^E79Q^ mutation was associated with radiation resistance, an effect more pronounced in immune competent mice [54]. Interrogation of the NRF2^E79Q^ tumors revealed significantly higher PMN-MDSCs and a shift from M1 to M2 macrophages relative to the NRF2-wild-type tumors [54]. The most robust effort to date, an analysis of over 1,000 tumors across 10 tumor subtypes using CPTAC proteogenomics data, identified that mutations in Keap1 and NRF2 were associated with diminished IFNγ and CD8 T cells [62]. Globally, there are disparities between tumor subtypes and immune related changes. Utilizing a pan-cancer transcriptional NRF2 signature and TCGA data, hyperactive NRF2 in NSCLC and other squamous cell carcinomas was associated with significant downregulation of IFNγ response genes and T-cell signaling [63]. On the contrary, this NRF2 signature did not have the same immunoevasive phenotype in other non-squamous cancer types including renal papillary carcinoma, hepatocellular carcinoma or bladder carcinoma [63].

## NRF2 activity in the immune microenvironment

In addition to tumor-intrinsic mechanisms, there is evidence that high NRF2 expression is deleterious to a cytotoxic anti-tumor immune response. Mechanistically, emerging evidence supports the significance of heme, a red blood cell metabolite, in the microenvironment in promoting NRF2-driven macrophage differentiation into immunosuppressive M2 phenotypes. Single-cell transcriptomics and functional assays have illuminated that NRF2-active macrophages commonly reside in hemorrhagic regions of tumors and that the presence of RBC-heme drives the formation of tumor associated macrophages (TAMs) supportive of tumor growth and invasion which have characteristically high NRF2 and HO-1 and low MHC-II [19]. In contrast to bone marrow–derived macrophages which are tumoricidal in the presence of IFNγ and anti-CD40, these heme-driven NRF2^hi^HMOX^hi^MHCII^lo^ TAMs are resistant to reprogramming by IFNγ or anti-CD40 [19]. Separate studies have defined that these HO-1^hi^ M2-TAMs are driven by NRF2 through HIF1α, p50 NF-κB–CSF-R1–C3aR axis signaling, tend to localize to the invasive margin, and support tumor, growth, angiogenesis and EMT transition [64, 65]. To further investigate the impact of these HO-1^hi^ M2-TAMs, Consonni et al. explored the immune microenvironment of tumors grown in HMOX1^fl/fl^;Lyz2-Cre mice and identified an increase in IFNγ^+^Granzyme B^+^ CD8 + T cells, effector memory T-cells, and an enhanced CD8 + /T_reg_ ratio in the mice deficient in HMOX1 [65].

In addition to macrophages, NRF2 expression has a detrimental impact on T cell proliferation and cytotoxicity. NRF2-knockout OT-1 T cells demonstrate enhanced activation upon exposure to ovalbumin and increased tumor suppression when adoptively transferred into ovalbumin-tumor bearing mice [66]. NRF2-knockout CD19 CAR T cells also exhibited greater in vivo expansion and tumor cytotoxicity [66]. This is further supported in mouse models of acute and chronic inflammation wherein KEAP1 suppression of NRF2 is critical for expansion of CD8 + T cells, metabolic reprogramming, and generation of stem-like T cells which enhance long term immunity [67]. Indeed, conditional knockout of KEAP1 drives NRF2 activity in CD8 + T cells and induces a PD-1^+^Tim-3^+^ terminally exhausted phenotype [68]. Recent evidence supports that NRF2 regulation of CD8 T cell fate is mediated through the prostacyclin receptor PTGIR and that silencing of PTGIR reverses terminal exhaustion and enhances effector responses [68].

## Impact of NRF2 on immunotherapy responses, TMB, PD-L1

Several biomarkers have been identified which predict tumor responsiveness to immunotherapy including PD-L1 immunohistochemistry staining, tumor mutation burden (TMB), predicted neoantigen load, mismatch repair deficiency, and pre-existing immune cell infiltration [69]. While PD-L1 upregulation in response to IFNγ expression and T-cell infiltration in the tumor immune microenvironment is a strong, positive biomarker for anti-PD1 monocolonal antibody responsiveness, a subset of tumors upregulate PD-L1 intrinsically in the absence of infiltrating immune cells and these tumors tend to be associated with both worse prognosis and resistance to anti-PD1 monotherapy [70]. Mouse allograft and patient derived xenograft studies in melanoma and NSCLC demonstrate that NRF2 activity promotes PD-L1 expression and diminishes the infiltration of lymphocytes into the tumor microenvironment [71, 72]. In a melanoma model, depletion of NRF2 by shRNA significantly enhanced the infiltration of CD4 and CD8 T-cells to the tumor, reduced the proportion of MDSCs and overall improved anti-PD1 responses [72]. Similar findings were noted in *KEAP1*^f/f^*/Pten*^f/f^ (K1P) mice, in which tumor bearing lungs had significantly diminished lymphoid cells and the infiltrating immune cells disproportionately expressed the PD-L1 immune checkpoint, although these tumors responded well to combination anti-PD1/anti-CTLA4 immunotherapy [71].

Two large database studies of NSCLC identified that KEAP1 mutations were associated with higher TMB and predicted neoantigens, but diminished response to immunotherapy [73, 74]^,^. This was also seen in a separate study of KRAS mutant NSCLC patients in which it was identified that a co-occuring mutation in KEAP1 or NRF2 was a significant and independent risk factor for resistance to immunotherapy [75]. A rigorous, unbiased study of lung adenocarcinoma patients utilizing single cell and traditional transcriptomics, proteomics, and immunohistochemistry sought to compare immunotherapy outcomes based on metabolic programming [76]. The authors identify distinct REDOX^high^ and REDOX^low^ classifications in which REDOX^high^ tumors were associated with mutations in KEAP1, NRF2, STK11, and SMARC4 which occurred in 73% of tumors compared to 17% of REDOX^low^ tumors. Relative to REDOX^low^ tumors, REDOX^high^ tumors were disproportionately lacking tumoricidal immune cells including CD4+ and CD8 + T cells and were instead enriched in pro-migratory fibroblasts and M2-macrophages. The clinical consequence of this REDOX signature was significant, with REDOX^high^ tumors exhibiting inferior objective response rates across two separate immunotherapy clinical trials- 39% vs 71% in one, and 17% vs 38% in the other, with corresponding worse progression free and overall survival as a result [76].

## Therapeutic opportunities to inhibit NRF2

NRF2 inhibitors are actively sought but remain clinically unproven. Efforts to inhibit NRF2 include both large-scale drug screens to identify direct and indirect inhibitors, as well as approaches to exploit novel NRF2-specific metabolic dependencies. A hallmark of NRF2-active tumors is metabolic reprogramming to promote glutamine metabolism, production of pentose phosphate pathway (PPP) intermediaries, and redox state effector proteins [77]. A CRISPR screen to identify redox vulnerabilities of KEAP1/NRF2-mutant tumors demonstrated hits in multiple enzymes in the pentose phosphate pathway, thioredoxin antioxidant system, glutathione reductase and mitochondrial superoxide dismutase 2 (SOD2) [78]. Other studies have shown that NRF2 KO models decreased transcription of numerous PPP enzymes including glucose-6-phsphate dehydrogenase (G6PD), 6-phosphogluconate dehydrogenase (PGD), transketolase (TKT), and transaldolase 1 (TALDO1) [77]. An important product of NRF2 signaling is the synthesis of glutathione, one of the most powerful ROS scavengers, to maintain cellular redox balance, detoxification, and promote cell proliferation [6, 79],. Shifting cellular priority to produce GSH requires that glucose and glutamine be redirected to anabolic pathways dedicated to GSH synthesis. This is accomplished through NRF2-driven upregulation of gamma-glutamylcysteine ligase (γGCL), a key enzyme in GSH production, GCLC and GCLM, the catalytic and regulatory subunits of γGCL, and xCT, an antiporter of cysteine and glutamate [80]. A transcriptomic and metabolomic analysis of NRF2-active NSCLC likewise found a shift to glutamine and cystine uptake and the production of purine nucleotides for glutathione synthesis at the expense of mitochondrial respiration [81]. While this metabolic shift enables NRF2-active tumors extraordinary capacity to withstand cytotoxic and inflammatory insults, the addiction of these tumors to glutamine metabolism provides a potent therapeutic target.

Early attempts to target glutaminase (GLS), the enzyme responsible for conversion of glutamine to glutamate, were limited by poor specificity, dose-limiting toxicity, and inefficacy. More recently, CB-839, a potent glutaminase inhibitor, demonstrated efficacy in preclinical trials of triple negative breast cancer and pancreatic cancer, two difficult to treat cancers with unique metabolic dependencies on glutamine consumption [82, 83]. This finding led to its investigation in biomarker selected NRF2-active tumors. In preclinical models of NRF2-mutant HNSCC, CB-839 enhanced radiation responsiveness [54]. Likewise, CB-839 is an efficacious radiosensitizer in KEAP1 mutant but not wildtype NSCLC [51, 84]. CB-839 is now under investigation in phase I/II clinical trials in a range of solid and hematologic malignancies (Table 2). Disappointingly, the KEAPSAKE trial, a Phase 2, randomized, multicenter, double-blind study of CB-839 with pembrolizumab and chemotherapy versus placebo with pembrolizumab and chemotherapy for first line treatment of metastatic KEAP1/NRF2-mutant NSCLC was terminated early due to lack of clinical efficacy. A related glutaminase synthase inhibitor, IACS-6274, demonstrated clinical efficacy in overcoming cisplatin-resistance in an NRF2-active HNSCC xenograft model [85]. This drug has now entered a phase 1 clinical trial in combination with chemotherapy in advanced/recurrent solid tumors with cisplatin resistance (Table 2). Alternative approaches to target metabolic dependencies include use of the PPP inhibitor, 6-aminonicotinamide, which demonstrated efficacy in Keap1^fl/fl^ tumor models [20].

**Table 2:** Active oncology clinical trials targeting the NRF2 pathway on clinicaltrials.gov.

NCT	Study title	Interventions	Drug mechanism	Phase	Conditions
NCT03872427	Testing Whether Cancers With Specific Mutations Respond Better to Telaglenastat Hydrochloride, BeGIN Study	Telaglenastat HCl	Glutaminase inhibitor	2	Advanced Solid Tumors
NCT04250545	Testing of the Anti Cancer Drugs CB-839 HCl (Telaglenastat) and MLN0128 (Sapanisertib) in Advanced NSCLC	Sapanisertib|Telaglenastat HCl	mTORC1/2 inhibitor, glutaminase inhibitor	1	Non-small Cell Lung Cancer
NCT05039801	IACS-6274 With or Without Bevacizumab and Paclitaxel for the Treatment of Advanced Solid Tumors	Bevacizumab|IACS-6274|Paclitaxel	anti-VEGF-A mAb; Glutaminase inhibitor, Tubulin inhibitor	1	Advanced Solid Tumors
NCT05275868	Study of MGY825 in Patients With Advanced Non-small Cell Lung Cancer	MGY825	unpublished	1	Non-small Cell Lung Cancer
NCT05580861	Sulfasalazine in AML Treated by Intensive Chemotherapy: Elderly Patients-first Line Treatment	Sulfasalazine	xCT inhibition	1 | 2	Acute Myeloid Leukemia
NCT05678348	Pyrimethamine as an Inhibitor of NRF2 in HPV-negative Locally Advanced Head and Neck Squamous Cell Carcinoma	Pyrimethamine	DHFR inhibitor	1	Head and Neck Cancer
NCT05954312	A First-in-Human Study to Evaluate the Safety and Tolerability of VVD-130037 in Participants With Advanced Solid Tumors	VVD-130037	KEAP1-NRF2 allosteric glue	1	Advanced Solid Tumors
NCT06620029	BMX-001 + Paclitaxel in Adult Patients With Advanced, Recurrent, Metastatic Ovarian or Endometrial Cancer	BMX-001 Paclitaxel	NFkB and HIF-1a	1 | 2	Ovarian, Endometrial Cancer

Another approach to drug discovery has been the implementation of chemical proteomics to map NRF2-reactive cysteine molecules in search for druggable targets. This approach led to the discovery that C274 of the orphan nuclear receptor NR0B1 has a key role in regulating the NRF2 program in KEAP1-mutant NSCLC [86]. In a separate, genome-wide CRISPR screen fructosamine-3-kinase (Fn3K), a kinase involved in protein de-glycation, is critical for NRF2 transcriptional function [87]. Absent Fn3K, NRF2 is glycated, a post-translational modification which enhances KEAP1-driven degradation, impairs NRF2 binding to transcriptional cofactors sMAF, reduces NRF2 binding in the promoter region of target genes, and leads to reduced transcription of canonical NRF2 pathway genes [87]. Therefore, Fn3k is a potentially actionable drug target and is under active investigation. Another chemoproteomics approach, led by Vividion Therapeutics identified a KEAP1 C151-reactive molecule which allosterically activates KEAP1, enhances the KEAP1:CUL3 interaction, and promotes NRF2 degradation [88]. This targeted approach yielded an exceptionally specific inhibitor, VVD-065, which demonstrated preclinical efficacy as both a monotherapy and radiation sensitizer and is currently in a phase I clinical trial (NCT05954312) [88].

Other approaches have included large scale drug screens for NRF2 inhibition. Masayuki Yamamoto’s lab identified halofuginone in a chemical library screen and demonstrated effective NRF2 suppression via suppression of prolyl-tRNA synthetase activity and amino acid starvation response [89]. Although effective in enhancing chemotherapy responses, halofuginone had notable toxicity due to its off-target effect on short-lived proteins and a follow-up study demonstrated that a micelle-encapsulation formulation of halofuginone was more readily tolerated with similar efficacy [90]. A related high throughput screen by Shyam Biswal’s lab identified ML385, a small molecule which directly interacts with the NRF2-MAFG complex and suppresses NRF2 transcriptional activity [91]. Similarly, a small molecule screen identified MSU38225 as an inhibitor of NRF2 which enhances NRF2 proteasomal degradation and sensitizes NRF2-active tumor cells to chemotherapy [92]. Three other small molecules, Brutasol, Luteolin, and AEM1, all have demonstrated efficacy in cell line and xenograft models to induce NRF2 suppression and sensitize NRF2 active tumors to chemotherapy [93–95],. Other nanoparticle approaches are earlier in development [96]. Finally, our team recently published the result of a cell-based screen which identified pyrimethamine and mitoxantrone as novel NRF2 inhibitors [97]. Pyrimethamine, an FDA-approved drug used in the treatment of protozoal infections, demonstrated efficient NRF2 suppression in both cell lines and in vivo in a K14Cre;LSL-NRF2^E79Q/+^ GEMM. For this reason, our team is conducting an early phase I window-of-opportunity biomarker clinical trial of pyrimethamine in locoregionally advanced head and neck cancer patients (NCT05678348, Table 2). Further exploration identified that mechanistically, pyrimethamine suppresses NRF2 activity through DHFR inhibition, and SAR optimization of pyrimethamine produced a novel DHFR inhibitor, WCDD115, with improved NRF2 potency currently undergoing preclinical exploration as a radiation sensitizing agent [98].

It is imperative, however, to caution that despite the enthusiasm for targeting NRF2 in cancer, there are potential risks involved, both locally and systemically. While constitutive NRF2 activity portends a survival advantage to cancer cells, data support that host NRF2 activity may also retain a protective role against tumor growth. For example, Keap1^fl/fl^ mice demonstrate an enhanced resistance to tumor metastases [46]. Correspondingly, mice with systemic deficiency of NRF2 have increased susceptibility to accelerated cancer proliferation and lung metastasis, in part through recruitment of MDSCs [46]. Therefore, one challenge in NRF2 targeted therapies is how to chemically target a critical cancer dependency while not inadvertently creating a host environment with increased susceptibility to cancer progression. This will require considerable attention to treatment duration, toxicity, and optimal combination approaches in clinical trial design. As NRF2 inhibitors progress in preclinical and clinical trials, acute and transitory NRF2 inhibition strategies should be considered to optimize the local and immunologic effect without creating an unintended susceptibility to distant metastasis.

## Conclusion

NRF2 activation is a common event during tumorigenesis and is associated with metabolic reprogramming and alterations in the immune microenvironment. A confluence of recent scientific evidence has illuminated how NRF2-driven transcriptional programs support cancer cell survival through tumor-intrinsic mechanisms and also sculpt the tumor immune microenvironment to suppress cytotoxic immune responses and dampen the efficacy of anti-PD1 checkpoint immunotherapy. Mechanistic insights from genetically engineered mouse models and clinical samples highlight the ability of NRF2 signaling to inhibit cell death pathways, impair STING and NF-kB signaling, and drive an immunosuppressive microenvironment which is favorable to tumor progression. These findings demonstrate that NRF2 activity may serve as both a clinical biomarker as well as an actionable therapeutic target. Future research should investigate opportunities to overcome NRF2-driven immunoevasion and sensitize tumors to treatment. NRF2 addiction provides an exciting therapeutic opportunity especially in the context of multimodality therapy and several promising small molecule inhibitors are currently in clinical trials. As the landscape of research on NRF2 widens, especially with respect to clinical trials, it will be critical to identify optimal therapeutic strategies and to define unwanted off-target effects.
